# The Mitochondrial Protein MitoNEET as a Probe for the Allostery of Glutamate Dehydrogenase

**DOI:** 10.3390/molecules27238314

**Published:** 2022-11-29

**Authors:** Chimere Nnatubeugo, Erica Johnson, Sarah Gisondi, Felicia Roland, Werner J. Geldenhuys, Michael A. Menze, Mary E. Konkle

**Affiliations:** 1 Department of Chemistry, Ball State University, 2000 W. University Avenue, Muncie, IN 47306, USA; 2 Department of Chemistry, Eastern Illinois University, Charleston, IL 61920, USA; 3 Pharmaceutical Sciences, School of Pharmacy, West Virginia University, Morgantown, WV 26506, USA; 4 Department of Neuroscience, School of Medicine, West Virginia University, Morgantown, WV 26505, USA; 5 Department of Biology, University of Louisville, Louisville, KY 40292, USA

**Keywords:** free fatty acids, beta-oxidation, mitochondria, green tea, hyperinsulinism

## Abstract

The proteins glutamate dehydrogenase (GDH) and mitoNEET are both targets of drug development efforts to treat metabolic disorders, cancer, and neurodegenerative diseases. However, these two proteins differ starkly in the current knowledge about ligand binding sites. MitoNEET is a [2Fe-2S]-containing protein with no obvious binding site for small ligands observed in its crystal structures. In contrast, GDH is known to have a variety of ligands at multiple allosteric sites thereby leading to complex regulation in activity. In fact, while GDH can utilize either NAD(H) or NADP(H) for catalysis at the active site, only NAD(H) binds at a regulatory site to inhibit GDH activity. Previously, we found that mitoNEET forms a covalent bond with GDH in vitro and increases the catalytic activity of the enzyme. In this study we evaluated the effects of mitoNEET binding on the allosteric control of GDH conferred by inhibitors. We examined all effectors using NAD or NADP as the coenzyme to determine allosteric linkage by the NAD-binding regulatory site. We found that GDH activity, in the presence of the inhibitory palmitoyl-CoA and EGCG, can be rescued by mitoNEET, regardless of the coenzyme used. This suggests that mitoNEET rescues GDH by stabilizing the open conformation.

## 1. Introduction

Glutamate dehydrogenase (GDH) catalyzes the conversion of glutamate to α-ketoglutarate and ammonia with the concomitant reduction of NAD(P) to NAD(P)H. The enzyme is evolutionarily well conserved and its oligomeric structure can be described as a homohexamer composed of a dimer of trimers [1]. GDH has three key structural portions; the first is the nucleotide binding domain (NBD) where both coenzymes bind. The second is the pivot helix that transitions GDH between its two predominant conformations, open and closed (also called abortive). The third structural portion, known as the antenna, is only present in higher order organisms (such as humans) [2]. The presence of the antenna leads to complex allosteric control to ensure that GDH performs an anaplerotic reaction to replenish α-ketoglutarate in the Krebs cycle. However, this simultaneously increases the concentration of free ammonium ion [2].

Guanosine triphosphate (GTP), palmitoyl-CoA, and the green tea polyphenol epigallocatechin gallate (EGCG) are known allosteric inhibitors of GDH [2]. The inhibition of GDH by GTP has implications in human health and disease. Mutations found in children with hyperinsulinemia/hyperammonemia (HI/HA) negate the negative allostery of GTP [3]. GTP binds at a regulatory site near the pivot helix of GDH [4]. EGCG has been promoted as a drug candidate for HI/HA as it could potentially restore allosteric inhibition that was lost due to mutations in the GTP binding site of GDH. EGCGs low toxicity and high bioavailability further strengthen the case for this approach [5].

Activation of GDH is accomplished by adenosine diphosphate (ADP) and the amino acid leucine [6]. ADP activation increases carbon flux through the Krebs cycle and, thereby, increase production of adenosine triphosphate (ATP) by oxidative phosphorylation (OXPHOS) [1]. The regulatory function of GDH activation by leucine is less obvious, but activation was achieved when a stable analog of leucine (b-2-aminobicycle(2.2.1)-heptane-2-carboxylic, BCH) was used [7]. However, activation by BCH was tightly coupled with increased glutaminolysis and insulin secretion [5].

The protein mitoNEET (E.C. 2.6.1.3) is a [2Fe-2S]-containing protein first discovered and heralded as a drug target in type-2 diabetes [8,9,10]. Surprisingly, despite different subcellular localizations at the mitochondrion, GDH and mitoNEET form a covalent complex via a disulfide bond between Cys319 (GDH) and Cys84 (mitoNEET) in vitro [11]. This bond increased the catalytic activity of GDH by 58% which can be negated by the addition of 2-mercaptoethanol [11]. Here, we elucidate how these two macromolecules interact with each other and determines the impact of mitoNEET on the complicated allostery of GDH. Allosteric inhibitors (palmitoyl-CoA, EGCG, and GTP), allosteric activators (ADP and leucine), and the co-substrates (NAD(H) and NADP(H)) were evaluated to find that mitoNEET binding had the largest rescue of GDH inhibition by EGCG. Additionally, the order of addition of these multiple components were varied to tease out any potential hierarchy of regulation. The rescue of GDH inhibition by palmitoyl-CoA led to the additional discovery that palmitoyl-CoA binds to mitoNEET with high affinity. Taken together, this study adds significant information for future drug discovery efforts targeting GDH and mitoNEET.

## 2. Results and Discussion

### 2.1. MitoNEET Rescues GDH from Palmitoyl-CoA Inhibition

GDH is an unusual dehydrogenase enzyme in that either NAD or NADP can be utilized as coenzyme. However, NAD(H) has two binding sites on GDH1, the catalytic and regulatory, whereas NADP(H) binds only to the catalytic site [12]. In each study, the order of addition of the coenzyme, allosteric ligand, and mitoNEET were varied (see Figures S1–S3.). Additionally, NAD was added at a high enough concentration to occupy both the regulatory and catalytic site.

**Figure 1:**
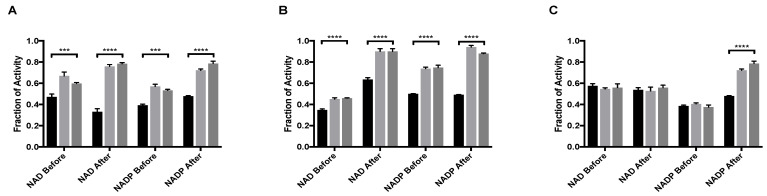
MitoNEET rescue of GDH activity from allosteric inhibitory ligands (**A**) Palmitoyl-CoA and (**B**) EGCG (**C**) GTP. In each graph the black bar is ligand alone, light gray bar is GDH + ligand + mitoNEET, dark gray bars are GDH + mitoNEET + ligand. The coenzymes NAD/NADP were added as designated along the x-axis. Fraction of activity is relative to the uninhibited GDH, error bars are S.E.M., and *** (*p* < 0.001), **** (*p* < 0.0001). Order of addition is depicted in Figures S1–S3.

MitoNEET caused the rescue of GDH activity from the inhibition by palmitoyl-CoA. When NAD was used as a coenzyme, the largest difference occurred between when NAD was added before mitoNEET + palmitoyl-CoA (0.60 ± 0.01 fraction recovered) versus after addition of mitoNEET + palmitoyl-CoA (0.78 ± 0.01 fraction recovered). This was a significantly larger recovery from 0.44 ± 0.04 percent recovery when GDH was treated with palmitoyl-CoA alone (Figure 1A). When considering NADP, there is a striking increase in recovery from 0.53 ± 0.02 when NADP is added first, to 0.79 ± 0.04 (Figure 1A) when NADP is added last. The order of addition, in terms of mitoNEET + palmitoyl-CoA or palmitoyl-CoA + mitoNEET had no significant impact on the rescue effect (Figure 1A). The binding site of palmitoyl-CoA on GDH is still unknown, making it difficult to infer structural underpinnings of these results. However, the significant increase in rescue if mitoNEET is bound before either coenzyme binds may indirectly indicate that mitoNEET stabilizes the open conformation of GDH and/or that palmitoyl-CoA hastens the loss of the [2Fe-2S] cluster from mitoNEET and allows the now available ligating cysteine residues to modify GDH as a mechanism of rescue [13].

### 2.2. MitoNEET Binds Palmitoyl-CoA with High Affinity

**Figure 2:**
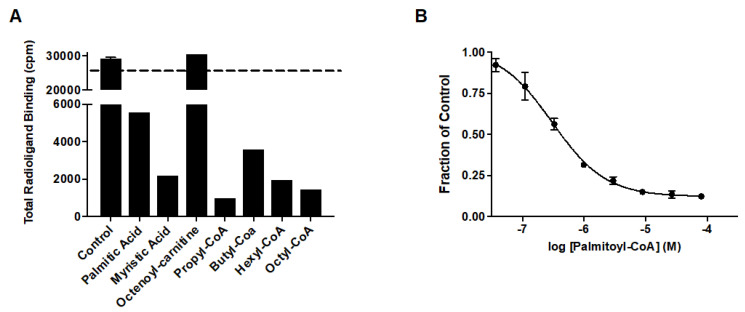
Free fatty acid and acyl-CoA binding to mitoNEET (**A**) Radioactivity competition assay using ^3^H-ATP binding to human recombinant mitoNEET. The dashed line represents 100% radioactivity recovered. Compounds screened at 100 mM. (**B**) Binding curve of Palmitoyl-CoA to mitoNEET. Error bars are S.E.M. N = 2.

**Figure 3:**
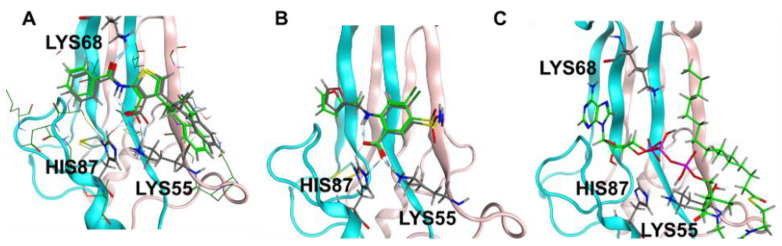
Docking studies of palmitoyl CoA binding to mitoNEET (**A**) RMSD determination of docking validation for M1 (7P0O.pdb) showing original ligand in grey color, and the docked ligand in green; (**B**) RMSD determination of docking validation for furosemide (6DE9.pdb) showing original ligand in grey color, and the docked ligand in green; (**C**) palmitoyl co-A docked into the binding pocket of mitoNEET.

Prior studies identified arachidonic acid as a ligand of mitoNEET with an IC_50_ of approximately 3 mM [14]. Furthermore, the glitazone compound PNU-91325 was shown to increase fatty acid synthesis, and knockout of mitoNEET affects lipid trafficking in cells [15]. Inspired by the result that mitoNEET rescues GDH activity from palmitoyl-CoA induced inhibition, a series of free fatty acids and acyl-CoA molecules were screened for binding to mitoNEET alone using a radioactive competition assay. The free fatty acids tested were palmitic and myristic acid and the acyl-CoA’s tested were propyl-CoA, butyl-CoA, hexyl-CoA, octyl-CoA, and palmitoyl-CoA. Binding affinity was observed at a concentration of 100 μM and compounds varied widely in their effect on ATP binding with propyl-CoA and myristic acid showing some of the highest effect in the competition assay (Figure 2A). A detailed binding curve was done for the most potent binding partner, palmitoyl-CoA, and determined an IC_50_ value for this fatty acid of 304 nM (Figure 2B). Docking studies were done next to evaluate the interaction between mitoNEET and palmitoyl-CoA. As seen in Figure 3, palmitoyl-CoA binds to the same binding pocket as furosemide and M1, which has been described previously [14,16]. Validation of the docking protocol indicated an RMSD < 2 Å for furosemide (RMSD = 0.458 Å) and M1 (RMSD = 0.680 Å), which is suggested to be acceptable in docking studies [17]. Key amino acid interactions included palmitoyl-CoA binding to LYS55, as well as with LYS68 correlate with those found for M1 and furosemide binding in the pocking of mitoNEET. Palmitoyl-CoA is of note physiologically because it can compete with ADP for the ANT transporter, thereby lowering substrate availability for OXPHOS [18]. This observation has developed into a molecular model that links obesity to diabetes and/or metabolic syndrome(s) [19].

### 2.3. MitoNEET Rescues GDH from Inhibition by the Natural Product EGCG

GDH is also inhibited by the natural compound EGCG, a polyphenol found in green tea. EGCG occupies the same regulatory site as NAD and prior work indicated that the binding of either molecule favored the closed, or abortive, conformation [19]. MitoNEET caused the rescue of activity of GDH from inhibition by EGCG. When NAD is added before either mitoNEET or EGCG, the recovery from inhibition is significant (*p* < 0.0001), but not impressive, with the fraction of activity of 0.35 ± 0.02 with no mitoNEET as compared to 0.45 ± 0.02 when mitoNEET is added. However, the difference in fraction of activity when NAD is added after mitoNEET and EGCG increased by 0.27 as compared to 0.10 when NAD is added before the EGCG. Additionally, introducing NAD after mitoNEET addition caused almost complete recovery (0.90 ± 0.03) of GDH activity despite the continued presence of EGCG (Figure 1B). The same effect was seen for NADP (Figure 1B), although the recovery when NADP was added first was twice that of when NAD was added first. This can be contributed to the closed confirmation when NAD occupies the regulatory site and thereby diminishes the rescue capacity of mitoNEET. NADP added after the ligand EGCG and mitoNEET resulted in the highest fraction of activity (0.94 ± 0.02) of any condition examined. No significant change in the amount of rescue was observed when the order of addition was changed from mitoNEET followed by EGCG to EGCG followed by mitoNEET, regardless of whether NAD or NADP was used. This indicates that in terms of power of allostery NAD bound to the regulatory site is most effective, that mitoNEET is stabilizing the open conformation, and that EGCG is occupying the regulatory site.

### 2.4. MitoNEET Rescues GDH from GTP Inhibition under Selective Conditions

GTP is the best understood inhibitor of GDH [4,13,20]. Mutation(s) in the GDH enzyme that negate the negative allostery of GTP and increase the activating allostery of leucine are known to cause the clinical condition of hyperinsulinemia/hyperammonemia (HI/HA). HI/HA is characterized by hypoglycemia and plasma ammonia levels that are 3–5 times higher than physiologically normal [20]. Due to the physiological relevance of understanding the inhibition of GDH by GTP to human health and disease, it is not surprising that a substantial amount of work has been done to understand the biochemistry and biophysics of this phenomenon [2,13]. GTP alone does not bind tightly to GDH, but works synergistically with NAD in the regulatory site to shift GDH to the closed, or abortive, conformation [13].

Strikingly, under only one assay condition the rescue of GDH activity from GTP inhibition by mitoNEET could be observed (Figure 1C). In order for rescue to occur, NAD could not be present at the regulatory site and mitoNEET needed to be present prior to the addition of GTP. Even with only NADP present, GTP binding prior to mitoNEET disallowed rescue of activity. The result that mitoNEET binding prior to GTP binding rescues GDH activity could indirectly imply that mitoNEET is stabilizing the open conformation and lessens GTP affinity at the regulatory site. There is no direct data at this time of this assessment. However, it is salient to note that the mitoNEET rescue from GTP depends upon both cofactor used and order of addition indicates more complex interactions are at play.

### 2.5. MitoNEET Enhances ADP Activation of GDH

**Figure 4:**
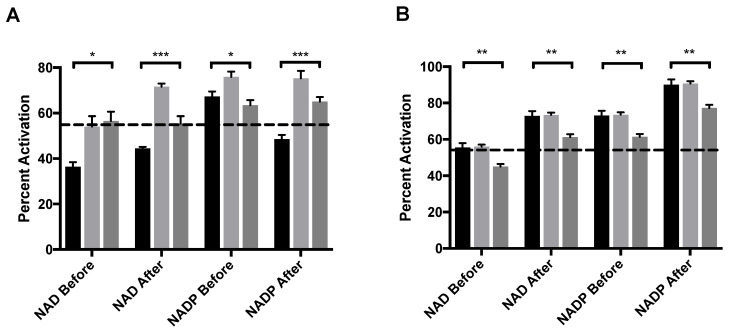
MitoNEET enhancement of allosteric activating ligands (**A**) ADP and (**B**) leucine. In each graph the black bar is ligand alone, light gray bar is GDH + ligand + mitoNEET, dark gray bar is GDH + mitoNEET + ligand. The coenzymes NAD/NADP were added as designated along the x-axis. The dashed line indicates the percent activation of GDH by mitoNEET alone. Percent activation is relative to the uninhibited GDH, error bars are S.E.M., and * *(p* < 0.1), ** *(p* < 0.01), *** (*p* < 0.001). Order of addition is depicted in Figures S1–S3.

ADP, an allosteric activator of GDH was also assayed with either NAD or NADP as a coenzyme. ADP binds at the NAD regulatory site [20]. The order of addition was varied to better elucidate the mechanism of activation. MitoNEET alone activates GDH in vitro by 58%, an increase that is mediated through the formation of a mixed disulfide bond as a novel GDH adduct [11]. When adding NAD before either mitoNEET or ADP, the percent activation does not rise above that of mitoNEET alone but is significantly higher than that of ADP alone regardless if ADP is present before (Figure 4A, light gray bar) or mitoNEET is added or not (Figure 4A, dark gray bar). However, if NAD is added after ADP and mitoNEET is present then a notable 72% activation occurs, but if NAD is added after (mitoNEET + ADP), the percent activation (56%) is the same as with mitoNEET alone. GDH appears to be more sensitive to activation by ADP when NADP is used as the coenzyme (67%) and present prior to ADP addition, so that the regulatory site is available to ADP. When NADP is added after the activators ADP and mitoNEET, the percent activation of 75% exceeds both that of ADP and mitoNEET alone. However, when NADP is added before the addition of mitoNEET and ADP only a 64% percent activation is observed, which is similar to the levels induced by ADP alone. Consistently, when mitoNEET is added prior to ADP then the percent activation is muted compared to when ADP is present prior to mitoNEET. These results clearly indicate that ADP binding to GDH prior to either coenzyme allows for enhanced activation by mitoNEET.

### 2.6. Leucine and mitoNEET Do Not Synergistically Activate GDH

Leucine and its stable analogue BCH activate GDH, but the binding site is unresolved for the mammalian enzyme [21,22]. For both coenzyme, addition of mitoNEET after the addition of leucine gave no impact in percent activation over the addition of leucine alone. When mitoNEET was added after leucine, it actually diminished the percent activation, regardless of the coenzyme. However, it did not fall below the threshold of percent activation by mitoNEET alone in most cases (Figure 4B). These results may indicate that mitoNEET and leucine use the same mechanism to activate the enzymatic activity of GDH, but without direct structural evidence for either partner it remains only a model that is supported by these results.

A recent publication allowed the examination of the structure of the ternary complex of bovine GDH-ADP-leucine for the first time (PDB structure 8AR7) [23]. Additionally, a potassium ion was co-crystallized in the structure and hypothesized to be an additional ligand. This work first identified the binding site for leucine at the interface of three different subunits allowing leucine to communicate conformation changes across the trimer protomers of GDH. This same work published a high-resolution structure of ADP alone (PDB structure 8AR7) and noted no significant structural changes as compared to the ternary complex. Our solution state results do differentiate between leucine and ADP, particularly when NADP is used as a redox cofactor.

## 3. Materials and Methods

### 3.1. Human MitoNEET Protein Expression and Purification

MitoNEET was expressed and purified as previously described from a bacterial system with a (cleavable) hexahistdine affinity tag [11]. MitoNEET was concentrated to 250 μM, stored at −80 °C, and used within 2 weeks of purification. The concentration of mitoNEET was determined using a UV-visible spectroscopy [11].

### 3.2. Bovine GDH Protein Preparation

Bovine GDH was purchased from Sigma-Aldrich (St. Louis, MI, USA). Prior to kinetic analysis, aliquots of GDH were dialyzed against 0.1 M sodium phosphate buffer at pH 8.5 using dialysis cassettes (ThermoFisher, Milwaukee, WI, USA) with a 10 kDa molecular weight cut-off. Two liters of sodium phosphate buffer was used in dialysis for approximately 2 h or until the milky white aliquots of GDH had visibly cleared. The freshly dialyzed GDH was then removed from the cassette and stored at 4 °C for at least 24 h before use.

### 3.3. Kinetic Analysis of GDH Activity Producing NADPH

The components of the assay (buffer, GDH, ligands, coenzyme, mitoNEET, and monosodium glutamate) were combined in in multiple different orders of addition. The assay sequences of the respective molecules are shown in Figures S1–S3. with the concentrations in Table S1. The kinetics of the reaction were followed by monitoring λ = 340 nm over 60 s with a step value of 0.5 s. The linear rate of NAD(P)H accumulation was calculated for each individual experiment. Coenzymes, ligands, and monosodium glutamate stock solutions were made immediately prior to use using reaction buffer (0.1 M phosphate buffer, pH 8.0). All reagents were purchased from Sigma-Aldrich (St. Louis, MI, USA).

All reactions were performed at a consistent total volume. In each case, the assay was started by combining GDH and reaction buffer. The amount of GDH added was adjusted such that the activity was at least 0.35 DAU_340_/min for assays that included the inhibitory ligands palmitoyl-CoA, EGCG, and GTP and between 0.15–0.30 DAU_340_/min for assays that included the activating ligands ADP or leucine. ADP stock solution were neutralized to pH 7 using 5 M KOH and aliquots were stored at −80 °C until use.

### 3.4. Data Treatment of Inhibitory Ligands

The rates observed in reactions with no allosteric inhibitor nor mitoNEET were considered an activity of 1.0. The rates of the samples that included the inhibitory ligands were divided by the uninhibited rates to calculate the fraction of activity. The statistical analysis (*p* values) was calculated using Prism 9.4.1. from GraphPad.

### 3.5. Data Treatment of Activating Ligands

The raw rates of reactions with no allosteric activator nor mitoNEET were considered the initial value. The formula (treated—initial)/initial × 100% was used to calculate percent activation. The statistical analysis (*p* values) was calculated using Prism 9.4.1. from GraphPad.

### 3.6. Radioligand Binding Assays

The radioactive binding assays were done as described previously. Briefly, human recombinant mitoNEET were incubated with SPA nickel-beads (PerkinElmer) for 30 min. The mitoNEET-SPA beads were then incubated with the compounds as well as 20 nM [3H]rosiglitazone (PerkinElmer) for 1 h, in 96-well format using a MicroBeta 2 (PerkinElmer) scintillation counter. Data analysis were done using Prism 6 (GraphPad).

### 3.7. Molecular Modeling Studies

Molecular modeling studies were done using MOE 2020.09 (ChemComp). The mitoNEET crystal structures with known ligands, furosemide (6DE9.pdb) [14] and M1 (7P0O.pdb) were used for the docking studies, since both ligands occupy the same binding pocket in mitoNEET. Palmitoyl-CoA structure was obtained from PubChem ID 644109. The QuickPrep method in MOE was used to prepare the protein for docking by adding the missing hydrogens and establishing the partial charges at pH 7.4. An induced-fit docking method was used to dock palmitoyl-CoA to mitoNEET, and the top 10 poses returned were evaluated for key interactions. Validation of the docking protocol was done by docking furosemide and M1 into the crystal structures, respectively and calculating the root-square-mean deviation (RMSD) between the original crystal and the docked pose. An RMSD < 2 Å is considered acceptable [17].

## 4. Conclusions

We show here for the first time that the [2Fe-2S] protein mitoNEET activates GDH through several strategies that include both amplifying the activation by ADP and leucine and by rescuing from the inhibitor GTP and palmitoyl-CoA. One interpretation of this indirect evidence is that mitoNEET stabilizes the open confirmation of GDH. This interpretation is supported by the large increase in rescue from EGCG inhibition when NAD is added after mitoNEET. The same pattern is seen for palmitoyl-CoA, the acyl-CoA thioester form of palmitic acid, but it is unknown where this ligand binds on GDH. Another interpretation could be that mitoNEET competes for the same regulating mechanism as NAD does. However, if that holds true then mitoNEET and ADP (which occupies the same regulatory site as NAD) would not be predsynergistic in their activation of GDH (Figure 4A). Additionally, this study yielded the novel findings that mitoNEET readily binds palmitoyl-CoA at nanomolar affinity. Both mitoNEET and GDH are targets for drug development efforts for metabolic syndromes, breast cancer, and neurodegenerative diseases. These biochemical findings will aid in these worthwhile efforts.

## Data Availability

The data presented in this study are available on request from the corresponding author.

## Supplementary Materials

The following supporting information can be downloaded at: https://www.mdpi.com/article/10.3390/molecules27238314/s1, Figures S1–S3: Order of Addition for GDH1 Kinetic Analysis; Table S1: Ligand Concentrations.

## Sample Availability

Samples of the compounds are available from the authors.
