# Supplementary material for: The Mitochondrial Protein MitoNEET as a Probe for the Allostery of Glutamate Dehydrogenase
Source: Molecules. 2022 Nov 29;27(23):8314. doi: 10.3390/molecules27238314 (PMC9737137; doi:10.3390/molecules27238314)
Supplement: Supplementary file 1 [file molecules-27-08314-s001.zip › molecules-1980964-supplementary.pdf]

molecules

Supplementary Materials

The Mitochondrial Protein MitoNEET as a Probe for the Allostery of Glutamate Dehydrogenase

Chimere Nnatubeugo [1], Erica Johnson [1], Sarah Gisondi [2], Felicia Roland [2], Werner J. Geldenhuys [3,4], Michael A. Menze [5] and Mary E. Konkle [1,2,*]

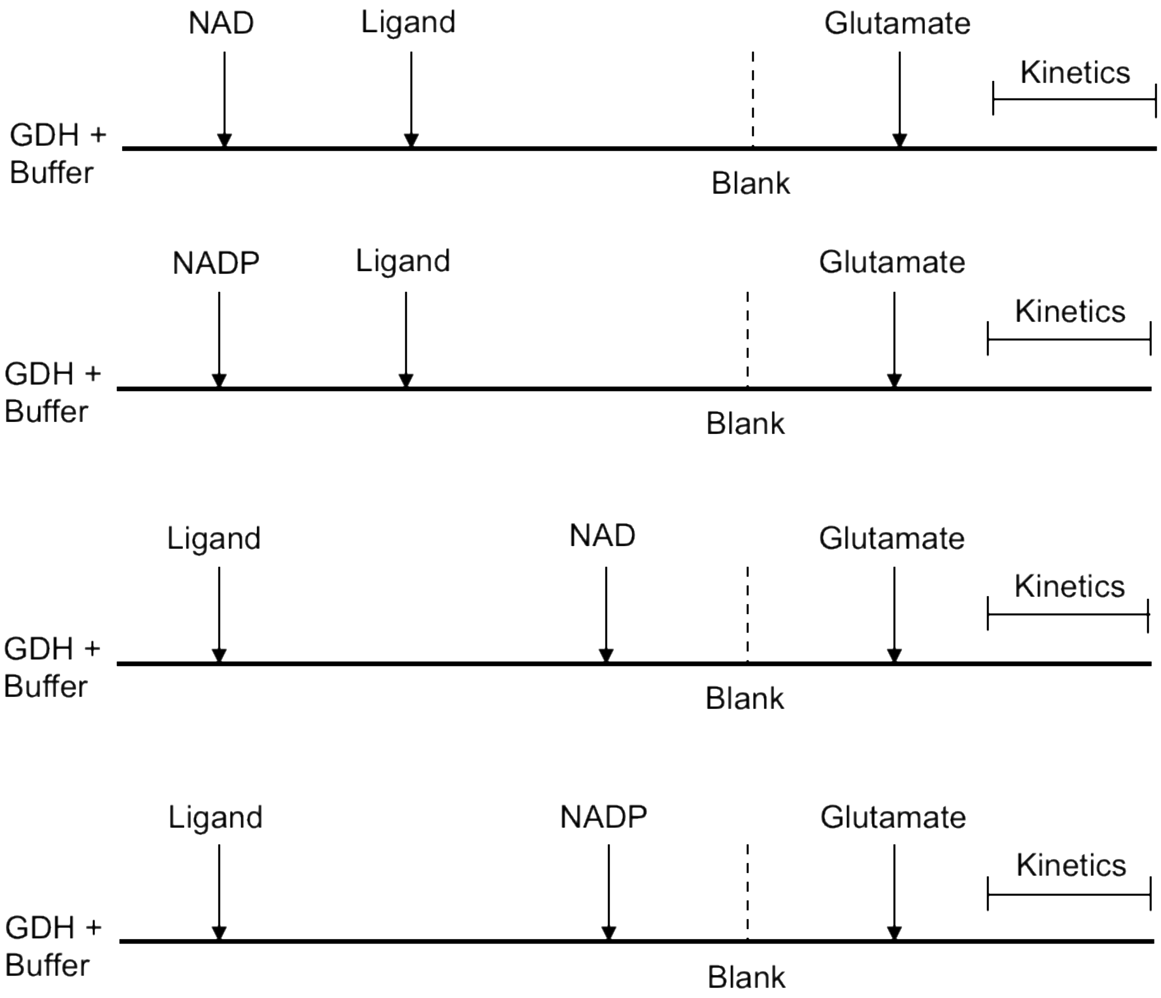

Figure S1. Order of addition for reagents for GDH kinetic analysis. The data from this assay is represented by the black bars in Figures 1 and 4.

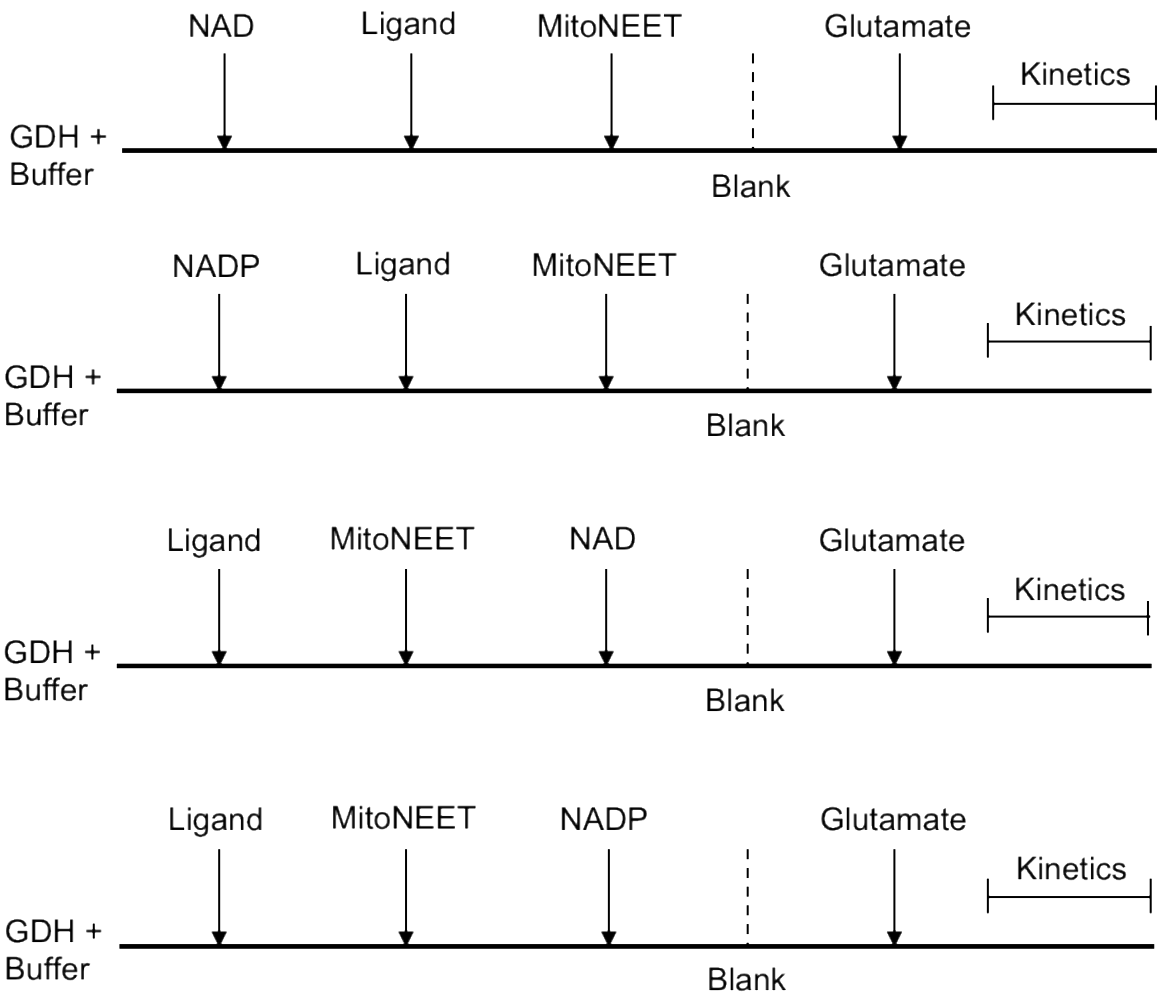

Figure S2. Order of addition for reagents for GDH kinetic analysis. The data from this assay is represented by the light gray bars in Figures 1 and 4.

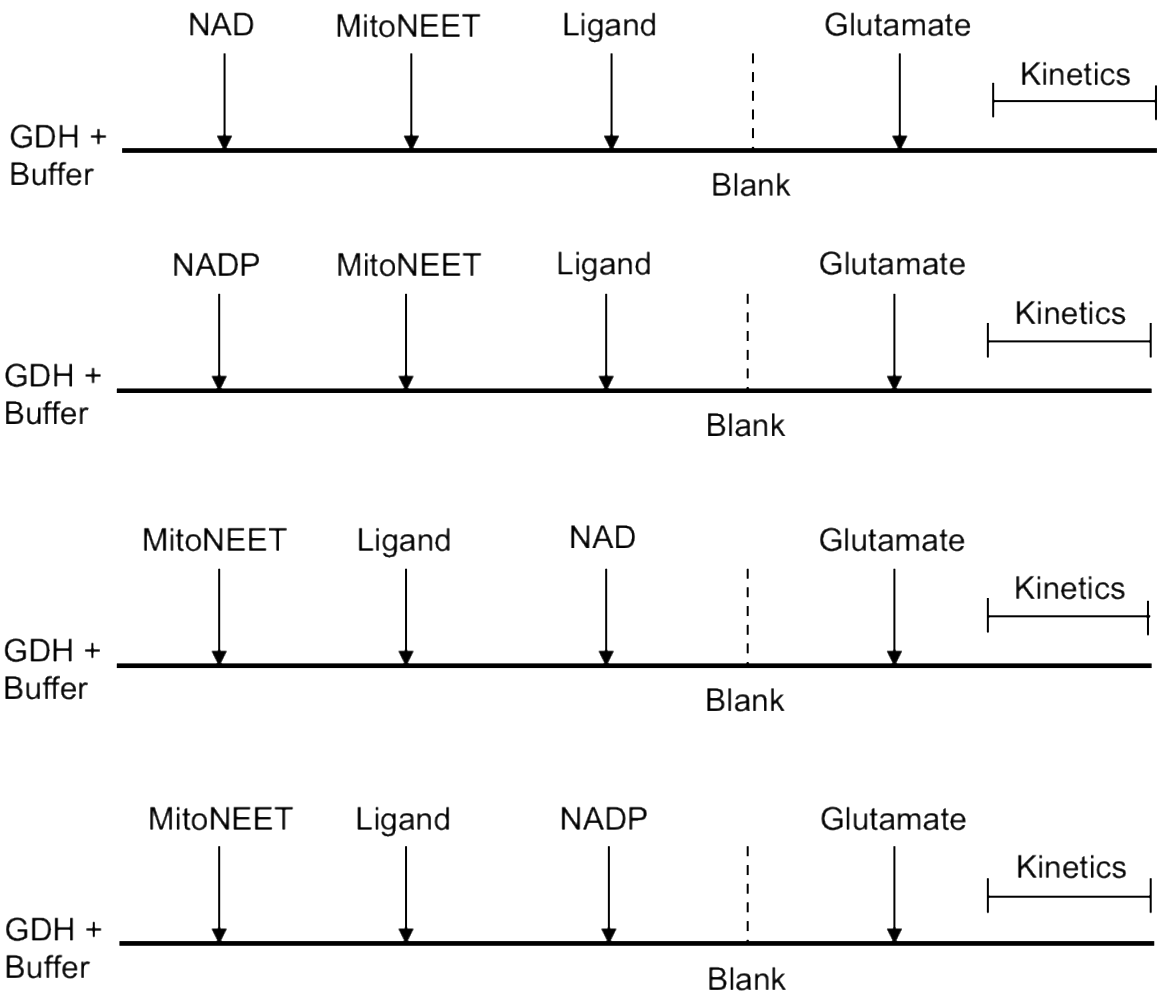

Figure S3. Order of addition for reagents for GDH kinetic analysis. The data from this assay is represented by the dark gray bars in Figures 1 and 4.

Table S1. The ligand concentrations used in the kinetic experiments [2, 5, 18, 19]. The final concentration of mitoNEET was 15 mM.

LIGAND	FINAL
GLUTAMATE	5.6 mM
NADP	2.67 mM
NAD^+	0.85 mM
ADP	11.3 mM
LEUCINE	15.6 mM
PALMITOYL-COA	1.8 mM
GTP	0.07 mM
EGCG	0.13 mM
